# Cost-effectiveness of ward-based pharmacy care in surgical patients: protocol of the SUREPILL (Surgery & Pharmacy In Liaison) study

**DOI:** 10.1186/1472-6963-11-55

**Published:** 2011-03-07

**Authors:** Monica de Boer, Maya A Ramrattan, Jordy JS Kiewiet, Eveline B Boeker, Kim B Gombert-Handoko, Nicolette AEM van Lent-Evers, Paul F Kuks, Marcel GW Dijkgraaf, Marja A Boermeester, Loraine Lie-A-Huen

**Affiliations:** 1 Department of Hospital Pharmacy, Academic Medical Centre, Amsterdam, The Netherlands; 2 Department of Surgery, Academic Medical Centre, Amsterdam, The Netherlands; 3 Department of Hospital Pharmacy, Onze Lieve Vrouwe Gasthuis, Amsterdam, The Netherlands; 4 Department of Hospital Pharmacy, Diakonessenhuis, Utrecht, The Netherlands; 5 Clinical Research Unit, Academic Medical Centre, Amsterdam, The Netherlands

## Abstract

**Background:**

Preventable adverse drug events (pADEs) are widely known to be a health care issue for hospitalized patients. Surgical patients are especially at risk, but prevention of pADEs in this population is not demonstrated before. Ward-based pharmacy interventions seem effective in reducing pADEs in medical patients. The cost-effectiveness of these preventive efforts still needs to be assessed in a comparative study of high methodological standard and also in the surgical population. For these aims the SUREPILL (Surgery & Pharmacy in Liaison) study is initiated.

**Methods/Design:**

A multi-centre controlled trial, with randomisation at ward-level and preceding baseline assessments is designed. Patients admitted to the surgical study wards for elective surgery with an expected length of stay of more than 48 hours will be included. Patients admitted to the intervention ward, will receive ward-based pharmacy care from the clinical pharmacy team, i.e. pharmacy practitioners and hospital pharmacists. This ward-based pharmacy intervention includes medication reconciliation in consultation with the patient at admission, daily medication review with face-to-face contact with the ward doctor, and patient counselling at discharge. Patients admitted in the control ward, will receive standard pharmaceutical care.

The primary clinical outcome measure is the number of pADEs per 100 elective admissions. These pADEs will be measured by systematic patient record evaluation using a trigger tool. Patient records positive for a trigger will be evaluated on causality, severity and preventability by an independent expert panel. In addition, an economic evaluation will be performed from a societal perspective with the costs per preventable ADE as the primary economic outcome. Other outcomes of this study are: severity of pADEs, number of patients with pADEs per total number of admissions, direct (non-)medical costs and indirect non-medical costs, extra costs per prevented ADE, number and type of pharmacy interventions, length of hospital stay, complications registered in a national complication registration system for surgery, number of readmissions within three months after initial admission (follow-up), quality of life and number of non-institutionalized days during follow-up.

**Discussion:**

This study will assess the cost-effectiveness of ward-based pharmacy care on preventable adverse drug events in surgical patients from a societal perspective, using a comparative study design.

**Trial registration:**

Netherlands Trial Register (NTR): NTR2258

## Background

Incidents caused by medications are a widely recognized issue in hospitalised patients. These incidents are known as Adverse Drug Events (ADEs), usually defined as 'any injury due to the use of medication' [1]. These may occur as an unavoidable result of the pharmacological action (side effect or Adverse Drug Reaction) or by the manner in which a drug is applied (medication error or substandard care). A preventable ADE (pADE) is defined as 'any injury caused by a medication error'. These pADEs are associated with substantial mortality and morbidity rates [2]. Besides clinical consequences, preventable ADEs have a considerable impact on health care costs [3,4].

A recent systematic review shows that 15.1% of all in-hospital adverse events (AEs), is medication related (i.e. ADEs) [5]. Current estimates show a large variance in the incidence of preventable ADEs--i.e. from 0.6 - 16 per 100 admissions [4,6-14], due to variability in population and variability in the sensitivity of detection methods. Surgical patients are typically at risk due to the transfer moments of surgical patients, medication changes before and after surgical intervention and a relatively frequent use of medications associated with a high prevalence of AEs, such as analgesics, anticoagulants and antibiotics [5,9]. The incidence of pADEs in surgical patients is unclear because of the absence of large studies using a standardized and general applicable detection method to determine pADEs in the surgical population.

In order to reduce the incidence and costs of in-hospital ADEs, several computerized strategies are developed, e.g. computerized order checking, barcode medication administration or computerized physician order entry (CPOE) with clinical decision-support system [10]. However, even in highly computerized hospitals, high rates of preventable ADEs remain [12]. Among health care professionals within the hospital organization, clinical pharmacists can fulfil a vital role in improving medication safety by face-to-face interaction with physicians and patients [15-17]. Instead of fulfilling the responsibility for the appropriate, safe and cost-effective use of medication from a central pharmacy, without patient contact or direct access to information on the clinical status of the patient and without face-to-face contact with the physician or nurses, nowadays active participation of pharmacists in the ward is common practice in countries such as the UK, USA and Australia [15]. This active participation of hospital pharmacists in the clinical process--also known as 'ward-based pharmacy care'--can reduce the occurrence of ADEs effectively with 66%-78% in medical and intensive care units [16,17]. Their activities consist of close review of medication at admission, active participation in multidisciplinary rounding teams, and counselling patients at discharge [15].

Only few studies have investigated the economic impact of the contribution of pharmacists to the clinical process [18-20]. Bond et al. studied the associations between clinical pharmacy services and total cost of care in approximately 1000 general surgery hospitals in the US. Clinical pharmacy services were associated with a cost saving per hospital per year of 5-8 million USD [18].

The departments of hospital pharmacy and surgery in the Academic Medical Centre in Amsterdam, The Netherlands, initiated the SUREPILL study: Surgery & Pharmacy in Liaison. The goal of this comparative study is to evaluate whether ward-based clinical pharmacy care can reduce (preventable) adverse drug events cost-effectively in elective surgical patients. The economic impact will be assessed from a societal perspective with incremental cost-effectiveness analyses of clinical pharmacy interventions against standard care. According to a recent systematic review there still is a need for comparative studies of high methodological standard measuring the effect of clinical pharmacy interventions in general [21]. Moreover, incidence of pADEs and preventability by a ward-based pharmacy team in surgical patients with more complex use of medication is not yet evaluated.

The main purpose of the SUREPILL study is to answer the following questions: 1) Will active participation of a clinical pharmacy team in surgical wards reduce preventable ADEs? 2) Will a clinical pharmacy team be cost-effective in reducing preventable ADEs in surgical wards receiving ward-based pharmacy compared to control wards receiving standard pharmaceutical care?

## Methods/Design

### Design

To answer the research questions, a multi-centre controlled trial, with randomisation at ward-level is designed. Participating centres are the Academic Medical Centre (AMC), the Onze Lieve Vrouwe Gasthuis (OLVG) in Amsterdam and the Diakonessenhuis in Utrecht. In each centre at least two surgical wards will participate. These study wards contain mainly gastro-intestinal and vascular surgical patients.

**Figure 1:**
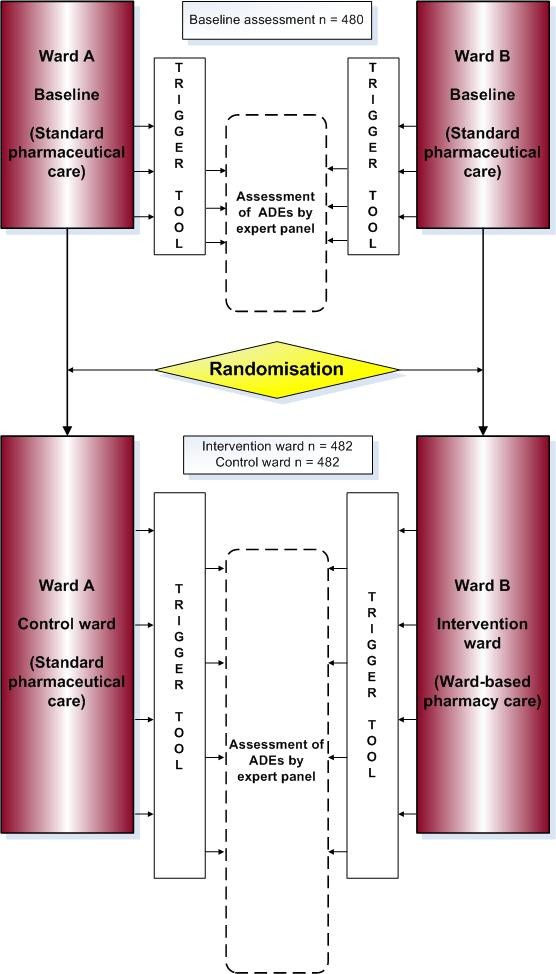
**SUREPILL study design and assessment**.

The study design is depicted schematically in figure 1. First, baseline assessments in each hospital in the participating surgical wards will be performed. Then, in each centre, one ward will be randomly assigned (one-time randomisation) as intervention ward, receiving ward-based pharmacy care, whereas the other ward(s) will serve as control ward, receiving standard pharmaceutical care from the central hospital pharmacy (i.e. current daily practice).

### Study population

Consecutive patients admitted to the surgical study wards for elective surgery with an expected length of stay of more than 48 hours will be included. Patients transferred from another hospital or from another ward within the hospital are excluded. Cross-over between the study wards will exclude the patient. Patients already included in the study will not be included for a second time in a following admission.

After evaluation by the Medical Ethics Committee (MEC) at the coordinating center (AMC Amsterdam) it has been decided that this study is exempt from ethical approval, because this study does not meet the criteria for Medical Scientific Research with humans under the Dutch Law (Dutch acronym: WMO). Informed consent was therefore not required. Furthermore, this protocol has been peer reviewed before study approval by the Dutch National Grant Committee of Health Care Efficiency Research (ZonMw grant 170882706).

### Intervention

#### Standard care

In all wards during baseline assessment and in control wards during the intervention phase, patients will receive standard pharmaceutical care from a pharmacy team in the traditional role of taking the responsibility for the appropriate, safe and cost-effective use of medication from a central pharmacy. This does not include patient contact or direct access to patients' medical records, nor does it include face-to-face contact with the ward doctors or nurses. The activities regarding individual patient pharmaceutical care consists of provision of community pharmacy data to the ward at admission, daily screening of alerts generated by the CPOE-system by the pharmacists during hospitalisation and provision of a discharge medication list for the community pharmacy.

#### Ward-based pharmacy care

A clinical pharmacy team will perform the ward-based pharmacy interventions. This team consists of pharmacy practitioners (pharmacy technicians, who completed additional training for ward-based pharmacy interventions) and hospital pharmacists.

Patients admitted at an intervention ward will receive bedside care from the ward-based pharmacy team. These interventions are tailored to cover critical steps in the medication process during the surgical pathway. On admission the pharmacy practitioner will perform medication reconciliation in consultation with the patient. This includes verification of the current use of community pharmacy medication. During hospitalisation the hospital pharmacist will review the medication charts daily and will optimise drug therapy when needed. The pharmacist combines information from CPOE-alerts, laboratory results and medical record information, in liaison with the ward doctor (face-to-face communication). At discharge the pharmacy practitioner will review the medication prescriptions by comparing them with the medication at admission. Unintended discrepancies will be discussed with the ward doctor. In addition, the pharmacy practitioner will perform patient counselling and send a complete list of discharge medication to the community pharmacy and general practitioner.

### Study outcomes and data collection

The primary outcome parameter is the number of preventable ADEs per 100 admissions. Secondary outcome parameters are severity of preventable ADEs, number of patients with preventable ADEs per total number of admissions, direct (non-)medical costs and indirect non-medical costs, extra costs per prevented ADE, number and type of pharmacy interventions, length of hospital stay, complications registered in a national complication registration system for surgery, number of readmissions within three months after the initial admission (follow-up), quality of life during follow-up and number of non-institutionalized days during follow-up.

To assess the incidence and severity of preventable ADEs, we will systematically evaluate patient records and the hospital information system by use of a developed and validated Trigger Tool. This tool is based on previously literature on trigger tools, such as the trigger tool for measuring harm in ICU patients [22], the trigger tool for detection of AEs in surgical patients [23] and the widely used Global Trigger Tool of the Institute for Healthcare Improvement [24,25]. This list of items, or triggers, are clues to identify potential pADEs. Previous studies have shown that a trigger tool can efficiently and consistently be applied to detect and identify preventable ADEs [26,27]. If patient records are positive for potential pADE triggers, an independent expert panel, consisting of senior hospital pharmacists and consultant surgeons, will assess the presence or absence of the potential pADE individually, blinded for the hospital and ward the patient was admitted to. They will determine the presence or absence by use of a causality assessment tool for surgical patients based on tools such as the Naranjo probability score and the WHO-UMC [28,29].

Besides causality assessment, the expert panel will also determine the severity of the ADE by use of a classification system called Common Terminology Criteria for Adverse Events (CTCAE) developed by the U.S. National Cancer institute [30]. This list is preferred, because a grading scale is provided for each AE term, in contrast to the National Coordinating Council for Medication Error Reporting and Prevention index (NCC MERP) [31]. Furthermore, the expert panel will assess the additional impact of each pADE to a patient's life, given his disease status. Discrepancies between the individual assessments will be discussed at consensus meetings of the experts.

For the economic evaluation, we will collect direct medical costs of all included admissions, such as diagnostic examinations, therapeutic interventions as well as the costs of monitoring by hospital pharmacists during hospitalization. Most data will be extracted from the hospital information system. Specifically, registration of tasks performed by the clinical pharmacy team will be done, not just as input values for the cost estimates, but also to support implementation decisions in anticipation of the program's cost-effectiveness. Moreover, we will gather information on direct (non-)medical costs, such as costs of out-patients care, out-of-hospital care and, if opportune, readmissions, as well as on indirect non-medical costs, such as productivity losses, by sending a patient questionnaire at three months following hospital admission. This questionnaire consists of an adapted version of the Health and Labour questionnaire developed by the Institute for Medical Technology Assessment in the Netherlands [32].

Besides cost-related questions, the validated EQ-5D questionnaire is used at three months after the initial admission to measure the quality of life of the patient [33]. The EQ-5D instrument contains five dimensions (mobility, self care, usual activities, pain/discomfort and anxiety/depression) for which patients have to indicate whether they experienced no, some or severe/extreme problems. In addition, patients have to assess their health status on a Visual Analogue Scale (VAS), ranging from zero for the worst imaginable health state to 100 for the best imaginable health state [33].

During the intervention period, we will document all interventions in the intervention wards performed by the clinical pharmacy team. In this study an intervention is defined as every change made in the patient's farmacotherapeutic management due to advice of one of the pharmacy team members. These interventions will be documented using a classification that is based on previous described types of interventions in literature [34]. The categories of this classification are listed in table 1.

**Table 1:** Types of interventions

Intervention	Including
Provision of drug information	physicians and nurses

Change route of administration	e.g., intravenous to oral

Drug dosage adjustment	sub- or supratherapeutic

Drug frequency adjustment	sub- or supratherapeutic

Recommendendation of monitoring	drug interaction, toxicology, allergy, adverse drug events

Suggest initiation of drug therapy	untreated indication

Recommendation of alternative drug therapy	formulary changes, drug interaction

Suggest discontinuing drug therapy	toxicology, allergy, adverse drug event, drug duplication

Others	not listed above

### Sample size and data analysis

The power calculation is based on the parallel comparison of the number of pADEs per 100 admissions between patients admitted at the intervention ward (with ward-based pharmacy care) and those admitted at the control ward (with standard care). We expect a mean base rate of five preventable ADEs per 100 admissions in the control group. This rate is conservatively estimated, based on the incidence of pADEs per 100 admissions in prospective observational studies in hospitalised patients (pooled mean 2.1%, range: 0.6%-16.2% [4,6-14]) and observed rates in control groups of intervention studies on the effect of clinical pharmacy on pADEs (pooled mean 11.6%, range: 11.4%-12% [16,17,26,35]). The difference in incidence rate between the observational and intervention studies can be predominantly explained by a higher sensitivity of ADE detection methods employed in the intervention studies.

In this study population, we hypothesize a relative reduction in pADEs of 60%. This hypothesis is based on the findings of two controlled studies assessing ward-based pharmacy in non-surgical wards, showing a reduction of 66% and a reduction of 78% [16,17].

With all admissions equally distributed over intervention and control wards, a sample of 964 admissions achieves 80% power at a 0.05 one-sided significance level to detect a reduction in the number of pADEs per 100 admissions from 5 to 2 or below (rate ratio <0.4), resulting from ward-based pharmacy care compared to standard care. To control for ward-specific ADEs at baseline, 480 patients equally distributed over the participating surgical wards, will be included at start. Hence, the total number of patients amounts to 1444 (480 + 964). This number of patients will be distributed over the participating centres according to their proportion of number of admitted patients in the wards.

With the total number of 1444 patients a Poisson regression of the number of pADEs with the type of treatment (intervention or control) as the main covariate of interest can be performed to detect a rate ratio below 0.4 with 80% power at a significance level of 0.05. This is performed while adjusting for other covariates like, for instance, hospital site, ward type, observation period, ADE levels at baseline, and patient age, even if these other covariates explain as much as 50% of the variance in pADE counts by type of treatment.

Because the number of pADEs per 100 admissions in the control condition is probably conservatively estimated, a recalculation of the needed sample size will be performed when the baseline pADE counts become available.

#### Economic evaluation

The economic evaluation of ward-based pharmacy care compared with standard care will be performed as a cost-effectiveness analysis from a societal perspective with the costs per preventable ADE as the primary outcome measure. Although fewer ADEs may result in reduced utilization of hospital resources, the savings may well be offset by increased pharmacy staff time. The time horizon of the evaluation will be three months following the initial admission to the surgery ward. This follow-up period will most probably include the full hospital admission as well as some time post-discharge. With this short time-span, costs and effects will not be discounted. Incremental cost-effectiveness ratios will be calculated, reflecting the difference in costs between ward-based pharmacy care and standard care relative to the difference in number of preventable ADEs.

Sensitivity analyses will be performed to account for sampling variability, plausible ranges of ward costs of various health care components, the substitution of pharmacists by pharmacy technicians and the weighing of preventable ADEs by level of severity based on expert opinion and using the CTCAE classification system. Furthermore, scenario analyses will be performed to account for differential allocations of ward-based pharmacy care during admissions. By monitoring the occurrence of preventable ADEs during admissions, ADE risk profiles over time (e.g. probabilities of events at day 1, day 2, etc.) can be constructed and used to develop scenarios for discontinuous or intermittent ward-based pharmacy care during admissions. Such scenarios may save costs which can be weighed against the risk of missing preventable ADEs.

Unit costs resources used will be derived from the most actual Dutch costing guideline for health care research [36]. The friction costs method will be applied to derive the costs of productivity loss due to sick leave from work [37]. After price-indexing all costs will be expressed in Euros for the base year 2010.

## Discussion

Measuring cost-effectiveness of clinical pharmacy is complex. Criteria for such research are not standardized. Recently, several recommendations have been stated in a systematic review [21], such as comparative design including a control group, providing details of clinical pharmacy interventions, considering health benefits, considering the effect of program factors (e.g. type of ward and hospital, and level of expertise of clinical pharmacist), considering applicability in other settings, taking a societal perspective when evaluating clinical pharmacy interventions, and including sensitivity and incremental cost-effectiveness analyses. The present study design meets these criteria.

For this study we choose a parallel design with preceding baseline assessment and with randomisation at ward level to assess the incidence of pADEs at baseline in the participating study wards and to determine the impact of the ward-based pharmacy care as well as to take into account the impact of differences between the observed study wards. Furthermore, a large number of factors are suggested to increase the risk of ADEs in hospitalised patients, such as drug characteristics (e.g. route of administration), patient-specific factors (e.g. number of medications taken) [9] and organisational factors (e.g. communication) [38]. These drug characteristics, patient-specific and organisational factors influence the incidence and severity of ADEs, but it is unknown to what extent. Therefore, the ideal situation to test the impact of a pharmacy team on clinical and economic outcomes would consist of an equal distribution of all these factors in experimental and control group. Straightforward comparison of two wards, with one ward only experimental and the other ward only control patients, implies the risk of unbalanced confounders because of the differences between the wards, patients and its staffing. By adding a baseline assessment it is possible to adjust for a priori differences between wards. In this manner a clear insight of the sole impact of the ward-based pharmacy care on pADEs is obtained.

We choose not to randomise at patient level within a ward because this would imply that one medical team provides care to both experimental and control patients concurrently. The drawback of this design is that the medical staff confronted with the pharmacy team has an increased awareness level of medication safety while they are treating control patients at the same time. This effect is know as the 'Hawthorne effect'--affecting the standard care in control patients [39]. In other words, experimental and control patients in one ward together implies a substantial risk of contamination of arms and thus blurring of effects on outcomes. Therefore, we choose a randomised design on ward level. Thus, in each participating centre, one ward is assigned as experimental (intervention) ward, whereas the other(s) will serve as control ward.

To reduce the risk of confounding, a spaced randomised design including 'cross over' would be desirable. Hereby each ward will serve as control and experimental ward separated with a wash-out period. However, this design was considered of limited value, because wash-out of learning experiences in the intervention arm at a ward during latter sequence might not be successful and the control period might thus become biased. Furthermore, this design is time consuming and may not be needed if extensive baseline assessments are done. Moreover, because of the long wash-out period needed, uncontrolled and unknown changes in factors associated with the risk of ADEs may have occurred.

The participation of three different types of hospitals, an university teaching hospital, a large teaching hospital and a community hospital, strenghtens the general applicability and effectiveness of ward-based pharmacy to various clinical settings.

This study also has its limitations. First, data collection for ADEs using the trigger tool method for surgical patients is retrospective. Information bias can play a role in the detection of preventable ADEs. Therefore, even though the trigger tool method is a standardised detection method, it may well underestimate the number of preventable ADEs. Secondly, to perform a cost-effectiveness analysis from a societal perspective, patients need to complete questionnaires to include data on health status and out-of-hospital use of resources. Without the need of an informed consent procedure as is the case in this study, the level of patient participation becomes less predictable. Finally, the ward-based pharmacy intervention has been standardized for the study, but work floor implementation may differ somewhat between different clinical pharmacy teams. Besides, the work of the clinical pharmacy team is partly performed by pharmacy technicians. Nevertheless, in many - certainly European - countries this may be easier to implement.

Although two studies have shown that decentralized clinical pharmacy care in the ward can reduce preventable adverse drug events [16,17], this study will provide new information to measure cost-effectiveness of clinical pharmacy care as well as a standardized strategy to intervene by a clinical pharmacy team. Searching for ways to improve patient safety in general and medication safety in particular remains focus of attention worldwide.

## Competing interests

The authors declare that they have no competing interests.

## Authors' contributions

MR, MD, MB and LL were involved in the development of the study proposal. KG and NvL are leading investigators at one of the participating hospitals. MdB, JK, MR, PK and MB are involved in developing the study methods. MdB, JK and EB are responsible for the data collection and analysis in all participating hospitals, MD specifically for the economic evaluation and data analysis. LL and MB are the study's principal investigators. MdB drafted the manuscript, all others contributed in reviewing the manuscript. All authors read and approved the final manuscript.

## Pre-publication history

The pre-publication history for this paper can be accessed here:

http://www.biomedcentral.com/1472-6963/11/55/prepub
